# Supplementary material for: 3D nitrogen-doped graphene foam with encapsulated germanium/nitrogen-doped graphene yolk-shell nanoarchitecture for high-performance flexible Li-ion battery
Source: Nat Commun. 2017 Jan 4;8:13949. doi: 10.1038/ncomms13949 (PMC5216101; doi:10.1038/ncomms13949)
Supplement: Supplementary Information — Supplementary Figures, Supplementary Table, Supplementary Notes and Supplementary References [file ncomms13949-s1.pdf]

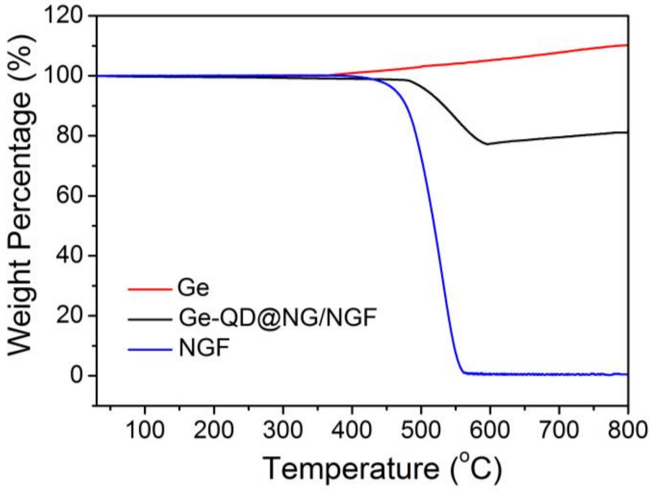

Supplementary Figure 1. Thermogravimetric analysis of Ge, NGF, and Ge-QD@NG/NGF. TGA curves of Ge, NGF, and Ge-QD@NG/NGF in air gas at a heating rate of 5 °C min^{-1}.

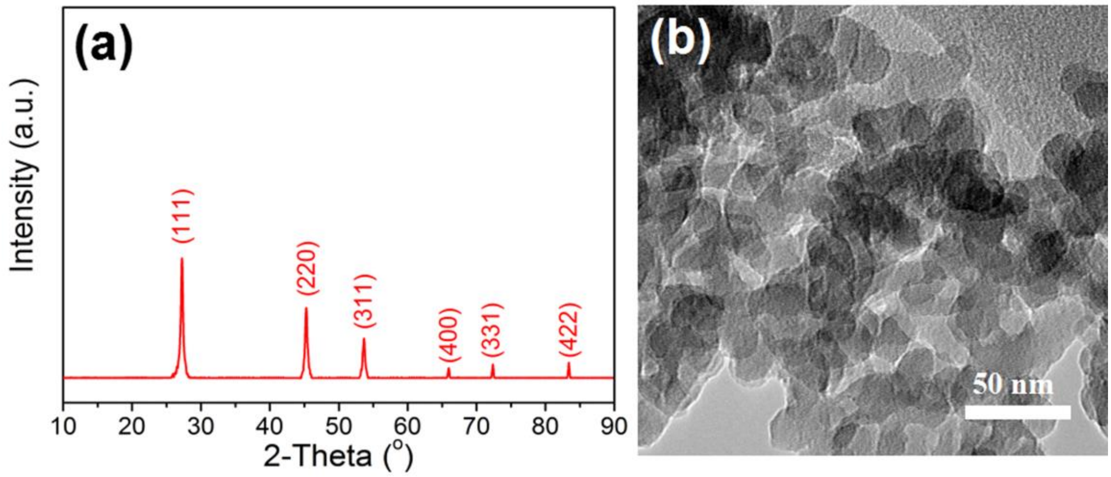

Supplementary Figure 2. Characterization of the Ge nanoparticle. a) XRD pattern and b) TEM image of the Ge.

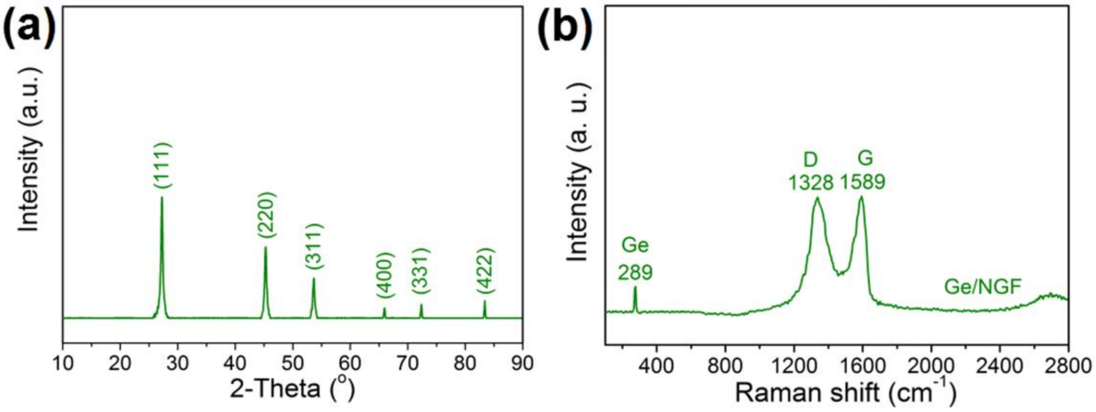

Supplementary Figure 3. Characterization of the Ge/NGF nanoarchitecture. a) XRD pattern and b) Raman spectra of Ge/NGF nanoarchitecture.

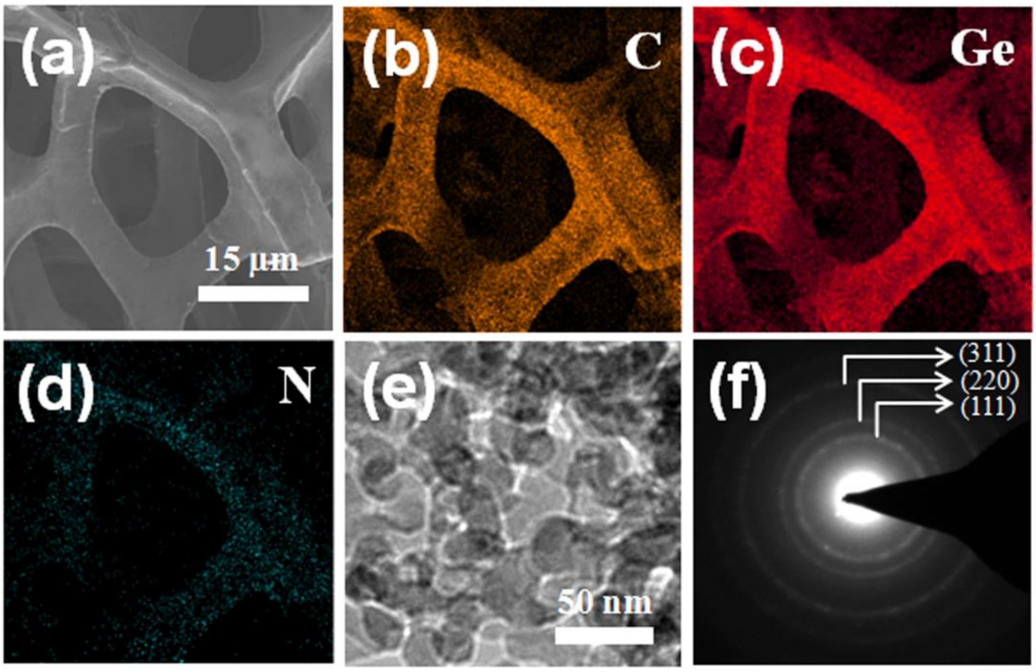

Supplementary Figure 4. Morphology characterization of the Ge/NGF nanoarchitecture. a-d) EDS elemental maps of Ge, C, and N, respectively. e) TEM image of the Ge/NGF nanoarchitecture. f) The electronic diffraction pattern corresponding to the Ge.

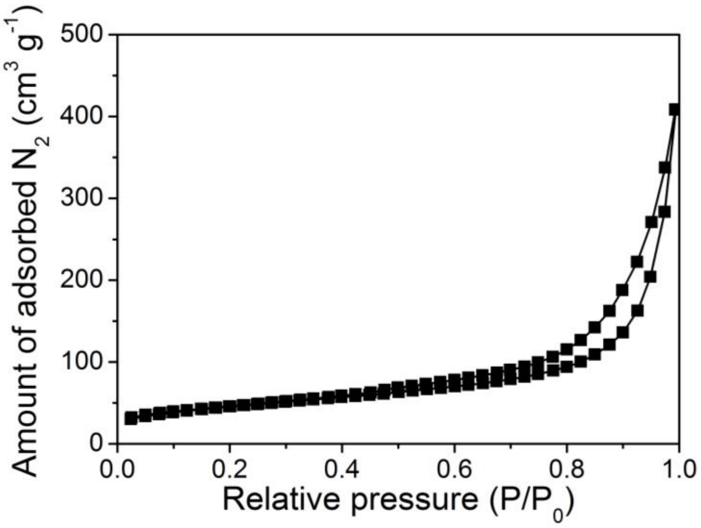

Supplementary Figure 5. Brunauer-Emmett-Teller characterization of the Ge-QD@NG/ NGF. Nitrogen adsorption/desorption isotherms of the Ge-QD@NG/NGF yolk-shell nanocomposite.

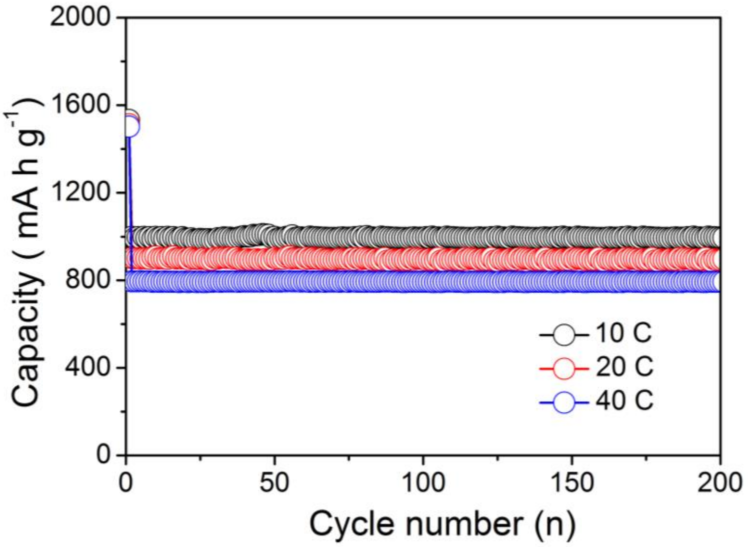

Supplementary Figure 6. Cycling evaluation of the Ge-QD@NG/NGF/PDMS. Cycling performance (discharge) of the Ge-QD@NG/NGF/PDMS yolk-shell electrode at 10, 20 and 40 C for 200 cycles.

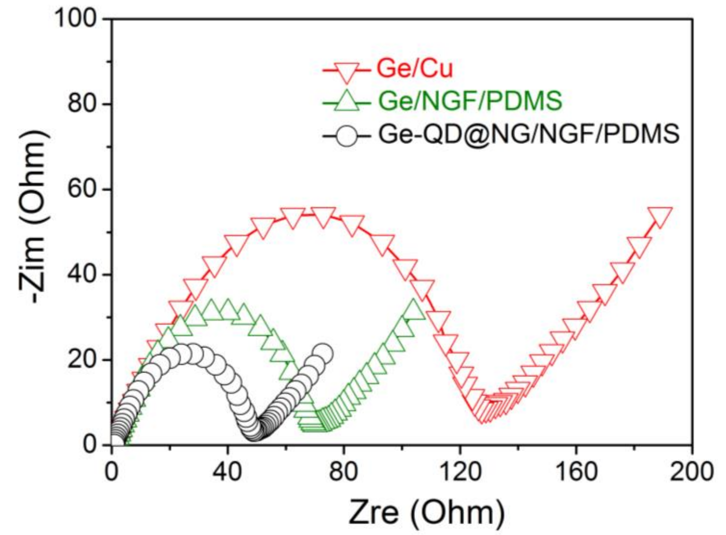

Supplementary Figure 7. Nyquist plots of the Ge-QD@NG/NGF/PDMS. Nyquist plots of the Ge-QD@NG/NGF/PDMS yolk-shell electrode (black), Ge/NGF/PDMS (green) and Ge/Cu (red) electrodes.

	NGF (wt%)	Ge (wt%)	Ge-NG/NGF(wt%)	X_{Ge}:X_{NG} (mass ratio)
600 ºC	0.06	105.12	77.22	73.44 : 26.56
625 ºC	0.04	105.74	78.07	73.82 : 26.18
650 ºC	0.03	106.39	78.58	73.85 : 26.15
675 ºC	0.03	107.05	79.07	73.86 : 26.14
700 ºC	0.03	107.13	79.11	73.84 : 26.16

Supplementary Table 1. Mass ratio analysis. Mass ratio of Ge and nitrogen-doped graphene at different temperature.

Supplementary Note 1

The residual weight percentage of the porous nitrogen-doped graphene foam (NGF), the pure Ge nanoparticle (Ge), and porous nitrogen-doped graphene foam with encapsulated Ge quantum dot@nitrogen-doped graphene yolk-shell nanoarchitecture (Ge-QD@NG/NGF) at 600, 625, 650, 675 and 700 °C are used to calculate the mass ratio of Ge and nitrogen-doped graphene (NG) in the Ge-QD@NG/NGF nanoarchitecture. The weight of Ge increased during high temperature zone because the formation of GeO_X, meanwhile the weight of nitrogen-doped

graphene decreased during the heat treatment owing to the decompose of nitrogen-doped graphene. Hence, we can use this equation to calculate the mass ratio of Ge and NG[1]:

$$W_{NG}\,(1–X_{Ge})+W_{Ge}X_{Ge}=W_{Ge\text{-}QD@NG/NGF}$$

Where W_{NG}, W_{Ge}, $W_{Ge\text{-}QD@NG/NGF}$ are the residual weight percentage of NG, Ge and Ge-QD@NG/NGF at different temperature, respectively, X_{Ge} is the mass ratio of Ge in the nanoarchitecture. The mass ratio is listed in the table S1, which is quite stable at different temperature, and the average mass ratio of Ge and NG in the nanoarchitecture is 73.76% of Ge and 26.24 % of nitrogen-doped graphene.

Supplementary References

1 Ji, J. Y. *et al*. Graphene-encapsulated Si on ultrathin-graphite foam as anode for high capacity lithium-ion batteries. *Adv. Mater*. **25**, 4673-4677 (2013).
